# Chemical and Microbial Leaching of Valuable Metals from PCBs and Tantalum Capacitors of Spent Mobile Phones

**DOI:** 10.3390/ijerph191610006

**Published:** 2022-08-13

**Authors:** Asma Sikander, Steven Kelly, Kerstin Kuchta, Anika Sievers, Thomas Willner, Andrew S. Hursthouse

**Affiliations:** 1 Department of Process Engineering, Hamburg University of Applied Sciences, Ulmenliet 20, 21033 Hamburg, Germany; 2 School of Computing, Engineering & Physical Sciences, University of the West of the Scotland, Scotland PA1 2BE, UK; 3 School of Health Life Sciences, University of the West of Scotland, Scotland G72 0LH, UK; 4 Institute for Environmental Engineering and Energy Economics, TUHH—Hamburg University of Technology, 21079 Hamburg, Germany

**Keywords:** bioleaching, tantalum, WEEE, acidophiles, waste management, green technology

## Abstract

We compared chemical and microbial leaching for multi-metal extraction from printed circuit boards (PCBs) and tantalum capacitor scrap. A mixed consortium of acidophiles and heterotrophic fungal strains were used in the experiments and compared to chemical leaching using specific acids (sulfuric, citric and oxalic acids). Under optimum conditions, 100% extraction efficiency of Cu, and nearly 85% of Zn, Fe, Al and Ni were achieved from PCB and tantalum capacitor scrap samples using sulfuric acid. The mixed consortium of acidophiles successfully mobilized, Ni and Cu (99% and 96%, respectively) while Fe, Zn, Al and Mn reached an extraction yield of 89, 77, 70 and 43%, respectively, from the PCB samples. For the tantalum capacitor samples, acidophiles mobilized 92% Cu, 88% Ni, 78% Fe, 77% Al, 70% Zn and 57% Mn. Metal mobilization from PCBs and tantalum capacitor scrap by *A. niger* filtrate showed efficient solubilization of Cu, Fe, Al, Mn, Ni, Pb and Zn at an efficiency of 52, 29, 75, 5, 61, 21 and 35% from PCB samples and 61, 25, 69, 23, 68, 15 and 45% from tantalum capacitor samples, respectively. Microbial leaching proved viable as a method to extract base metals but was less specific for tantalum and precious metals in electronic waste. The implications of these results for further processing of waste electronic and electrical equipment (WEEE) are considered in potential hybrid treatment strategies.

## 1. Introduction

Metals are the heart of a country’s economy. A steady supply chain of metals independent from geopolitical instabilities is essential for the national and economic security of a country. Transitioning to a green energy economy leads to an ever-growing demand for rare and critical metals that can lead to a shortage of supply from primary deposits. This has resulted in intense interest in developing efficient recycling techniques to recover these metals from suitable secondary sources. The end-of-life waste electric and electronic equipment (WEEE) offers potential as a source of these metals [1,2]. However, efficient recovery, viable economic conditions and eco-friendly recycling methods are needed. This is due both to the potential value of a variety of components of printed circuit boards (PCBs) and the relative concentrations of toxic and hazardous substances that need proper disposal. In a study by D’Adamo et al. [3], high-grade waste PCBs when totally recycled can give a net present value of around EUR 63 million. However, due to the heterogeneous mixtures of metals and materials in PCBs, recovery of the target metals is a technical challenge. Traditional recycling technologies such as mechanical, hydro and pyrometallurgical approaches were widely applied to recover many elements but cannot effectively target rare earth elements (REEs), which usually end up as dust or trapped in a slag phase [4]. Mechanical treatment involving physically dismantling, crushing and separation processes is widely used in recycling plants worldwide for pre-processing of materials which are then subject to further processing. Hydro and pyrometallurgical methods are the most commonly used techniques to recover base metals from WEEE [5,6]. These techniques involve either large volumes of highly corrosive leaching agents (hydrometallurgical processes) or in the case of pyrometallurgical processes high energy consumption, dust generation and emission of combustion gases [7,8,9]. In addition, other metal recycling techniques such as supercritical fluid, ionic liquid and physical separation are also gaining interest in the scientific community but efficient and suitable measures and techniques for the management of PCBs are elusive.

Bio-hydrometallurgy is a rapidly emerging eco-friendly technology that applies metal cycling mechanisms identical to biogeochemical cycles [10,11]. In bio-hydrometallurgical methods, this includes microbial leaching, bioreduction and biosorption, where metals are recovered from waste material by means of environmentally safe biogenic lixiviant secreted by microorganisms. These methods are not only cost-effective but their operational flexibility and selectivity towards metals make them quite promising for metal recycling [12]. Although the role of microorganisms in metal recycling is still in its infancy [13,14], the proven efficacy of biological methods in metal extraction from primary sources is the main impetus for defining its role in recycling and recovering critical elements from WEEE.

The growing demand for and criticality of tantalum supply presents a stimulus for methods of recycling. This research explores the chemical and microbial leaching possibilities of tantalum from spent capacitors using green reagents. Tantalum consumption is dominated by its use in capacitors in PCBs for electronic equipment with a typical tantalum capacitor containing 48–49% by weight of tantalum [15]. Despite being a rich source of critical metal, end-of-life tantalum capacitors are destined for landfill. Recycling tantalum is difficult due to challenges in its leaching and subsequent separation from other metals [16]. Currently, tantalum leaching involves highly corrosive and toxic solutions of concentrated HF [17,18,19], which highlights the need for an efficient and eco-friendly recycling process. 

Hydrometallurgical processes are technically mature but the economics of processing and the ecological impact, in particular of using toxic reagents, especially in the recovery of precious and rare metals, is still an issue [1]. The aim of this study is to find an environmentally friendly metal leaching technique from electronic waste. Consequently, we investigated the possibility of microbial extraction of valuable metals from PCBs and tantalum capacitor scrap as a potential alternative to conventional chemical leaching. We also investigated the potential of organic acids as alternative metal leaching reagents. A comparative study between chemical and microbial leaching methods was been carried out. The main parameters of pulp density, pH, reaction temperature and time were studied and optimized to evaluate efficiency, cost-effectiveness, feasibility and environmental impact. Potential recovery of minor metal content from WEEE and tantalum capacitor scrap and commercial viability of processes were also studied. For microbial leaching, a mixed consortium of iron- and sulfur-oxidizing acidophiles (*Acidithiobacillus ferrooxidans*, *Leptospirillum ferrooxidans* and *Acidithiobacillus thiooxidans*) and organic acid-producing heterotrophs (*Aspergillus niger*) were employed in comparison with specific acids (sulfuric, citric and oxalic). Studies show that the use of a mixed culture of bacteria is a better option than single microorganisms in metal leaching phenomena as different microorganisms can have distinct metal tolerance, which can result in a greater metal leaching capacity [20,21], whereas *Aspergillus niger* was reported for the recovery of precious metals from e-waste [22]. For metal dissolution by microbial consortia of iron and sulfur oxidizing acidophiles, the microorganisms follow thiosulfate and/or polythiosulfate pathways [2], biogenic sulfuric acid and ferric iron act as lixiviants and metals are mobilized to their ionic state in aqueous solution by proton attack via the formation of acids (acidolysis) or oxidation/reduction reactions (redoxolysis). In simplified form, this chemical reaction can be written as

(1) $$MS+H_{2}SO_{4}+\frac{1}{2}O_{2}\to MSO_{4}+S^{0}+H_{2}O$$

(2) $$S^{0}+\frac{3}{2}O_{2}+H_{2}O\to H_{2}SO_{4}$$

Here, *M* can be *Cu*^0^, *Zn*^0^, *Al*^0^, *Ni*^0^, *Fe*^0^, etc. 

However, heterotrophs need additional energy sources (glucose/sucrose) to grow and produce biogenic acids (lactic, oxalic, citric and gluconic acid in different concentrations), amino acids and metabolites [23]. These biogenic organic acids coupled with biogenic chelators can be efficiently used to release rare and critical metal ions from e-waste [24].

(3) $$C_{6}H_{8}O_{7}\;↔\;{\left(C_{6}H_{5}O_{7}\right)}^{3-}+3H^{+}$$

(4) $$CuO+2H^{+}\to \;Cu^{2+}+H_{2}O$$

Strong regulations on environmental pollutants and restrictions on illegitimate recycling practices create a high demand for novel green technologies to recover metals from waste. Efficient recycling of WEEE will not only address the environmental issues and supply risk of metals but also create job opportunities for biotechnologists, metallurgists, and skilled workers. Additionally, biotechnological recycling of WEEE in a circular economy approach can contribute to resource extraction from waste without having any related harmful impact on the environment [25]. 

## 2. Materials and Methods

### 2.1. Material Characterization

Electronic waste in the form of PCBs and tantalum capacitor scrap from spent mobile phones was used for the leaching experiments. PCBs and visually identified (VICs) surface mounted device (SMD) capacitors from spent phones were dismantled manually and ground in a cutting mill (Retsch cutting Mill SM300, Haan, Germany) to two particle size fractions (0.5 mm and 0.75 mm). For metal analysis, samples were digested using a Mars 6 Microwave Accelerated Reaction System (CEM corporation, Mathews, NC, USA). Two different digestion trials were performed, in the first trial, 0.1 g samples of milled PCBs and tantalum capacitors were mixed with 6 mL HCl (35% *v*/*v*) and 2 mL HNO_3_ (69% *v*/*v*). In the second trial, tantalum capacitor scrap samples were mixed in an acid mixture of 6 mL HCl (35% *v*/*v*), 2 mL HNO_3_ (69% *v*/*v*) and 2 mL HF (40% *v*/*v*). All samples were subjected to microwave-assisted digestion. The digested solutions were then filtered using a 0.45 µm Syringe Filter. The base metals (Al, Cu, Fe, Ni, Mn, Pb and Zn), precious metals (Au and Ag) and critical metal (Ta) were analyzed by inductively coupled plasma-optical emission spectroscopy (Agilent 5100 ICP-OES, Santa Clara, CA, USA). The validation of the ICP-OES analysis is provided in the Supporting Documents Table S1. 

### 2.2. Microorganism and Culture Conditions

Acidophilic bacterial strains: A mixed culture of acidophiles, consisting of iron- and sulfur-oxidizing strains such as *Acidithiobacillus ferrooxidans*, *Leptospirillum ferrooxidans* and *Acidithiobacillus thiooxidans* was used for microbial leaching studies. In order to activate the microorganisms, 10 mL of the stock bacterial culture was added to 90 mL of fresh modified Silverman and Lundgren medium (9K) containing (g/L): (NH_4_)_2_SO_4_ (2.0), KCl (0.1), MgSO_4_∙7H_2_O (0.25), KH_2_PO_4_ (0.5), S (1.0), supplemented with FeSO_4_∙7H_2_O (44.2). The medium was modified to promote the growth of iron- and sulfur-oxidizing species. The pH was set to 1.8 with H_2_SO_4_ (10% *v*/*v*). Since autoclaving of Fe^+2^ containing solutions leads to precipitation of iron therefore all medium ingredients except ferrous sulfate and the samples were sterilized. Sterile experiment set-up was achieved by autoclaving at 121 °C for 30 min at 15 psi in an autoclave. The flask was agitated at 150 rpm using an orbital shaking incubator (Gallenkamp Orbital Shaker—Incubator, Cambridge, UK) operated at 30 °C for 24 h. The activity of bacterial strains was monitored indirectly by measuring pH in the bioleaching medium which drops steadily with the passage of time. The pH was measured using a Thermo Scientific Orion DUAL STAR^TM^ pH/ISE meter. Following complete iron oxidation, the fully active culture was used for microbial leaching studies.

For the microbial leaching of tantalum capacitors, the heterotrophic fungus *Aspergillus niger* (DSM-2182) was employed. Fungal cultures were grown in medium containing (g/L): MgSO_4_ (0.03), NaCl (0.05), KH_2_PO_4_ (0.05), K_2_HPO_4_ (0.15), KCl (0.5) and sucrose (2.0). The pH of the medium for cultivation is set to 5.6 ± 0.1 using HCl (recommended by the supplier). Medium was sterilized (autoclaved at 121 °C for 30 min at 15 psi) before cultivation. A portion of active culture was suspended in the sterile medium and the flask was subjected to rotation for 48 h at 150 rpm and 30 °C. Following complete growth of the culture it was filtered and this filtrate as well as active *A. niger* spores were used for leaching experiments. Growth of fungal strains was monitored by measuring optical density (OD) at 600 nm.

### 2.3. Metal Leaching Experiments

#### 2.3.1. Leaching with Organic and Inorganic Acids

Solubility of metals from waste PCBs and tantalum capacitors were studied using organic acids namely citric acid (0.05 M) and oxalic acid (0.05 M) and inorganic acids H_2_SO_4_ and HNO_3_. H_2_O_2_ was used as the oxidizing agent with H_2_SO_4_. The different organic and inorganic acids were selected not only to compare the microbial leaching efficiencies with their specific acids but also to investigate the potential solubilization of metals from electronic waste employing organic acids in comparison with inorganic acids to assess the potential for lower toxicity reagents in acid leaching. Experiments were carried out in 250 mL Erlenmeyer flasks with a working volume of 100 mL, using a rotary shaker (Gallenkamp Orbital Shaker—Incubator). Pulp density of 10% and particle size of 0.75 mm of PCBs were used in the experiments.

Trials with organic acids were carried out at 30 °C and 150 rpm for 20 days. Every 5th day pH was measured and 1 mL of samples were taken for analysis.

In leaching experiments with inorganic acids, H_2_SO_4_ was used in a concentration range of 1.2–2 mol/L, whereas the quantity of H_2_O_2_ mixed with H_2_SO_4_ was in the range of 0.2–1.5 mol/L. The acid concentration was selected on the basis of previous studies [26,27]. Leaching was carried out at temperature range of 65–85 °C for 8 h. The flasks were agitated on a rotary shaker at a speed of 500 rpm. Then, 1 mL of samples were taken every 60 min. Leaching trials with HNO_3_ were conducted at a concentration of 1–4 mol/L. The agitation speed was set to 400 rpm and leaching was carried out at temperature range of 70–90 °C for 2 h. Then, 1 mL samples were taken every 60 min. At the end of the experiments, the flasks were harvested to obtain the leached residues and the final leached liquor. The residue collected was filtered (125 Ø cellulose filter, Whatmann, UK) and left to dry overnight. The leach liquor was analyzed for metal concentration. If not stated otherwise, all experiments were replicated in three runs.

#### 2.3.2. Microbial Leaching Studies

Microbial leaching experiments using acidophilic bacterial species (*Acidithiobacillus ferrooxidans*, *Leptospirillum ferrooxidans* and *Acidithiobacillus thiooxidans*) were carried out in 250 mL Erlenmeyer flasks, using a rotary shaker at 150 rpm and 30 °C (Gallenkamp Orbital Shaker—Incubator). Microbial leaching trials on PCBs were carried out at varying pulp density (2, 5, 7, 10%, *w*/*v*), particle size (0.5 and 0.75 mm) and initial ferrous iron concentration (1, 3, 5, 7 g/L) in 100 mL microbial leaching medium containing actively growing cultures. A 2% pulp density and varying particle size (0.5 and 0.75 mm) was used for microbial leaching trials on tantalum capacitor scrap samples. One variable at a time experiment was adopted to optimize the leaching process parameters and to assess how much a single change affected the result. The leaching process was monitored by checking pH, ORP (oxidation-reduction potential) and ferrous iron concentration of the leachate solution periodically. Ferrous iron concentration was measured by O-phenanthroline method [28]. The changes in system pH and ORP (mV)] were measured through the Thermo Scientific Orion DUAL STAR^TM^ pH/ISE meter. Every second day, 1 mL of the liquor was taken from each flask for metal content determination using a syringe (0.45 µm Syringe Filter). Any loss of volume of the microbial leaching medium due to evaporation and sampling was compensated with sterilized water. The system pH was maintained at 1.8 as per requirement using H_2_SO_4_ (10% *v*/*v*). Each flask experiment was completed in triplicate. Control experiments were carried out with non-inoculated microbial leaching medium. The experiments were performed for 10 days and the flasks were harvested to obtain the leached residues and the final leached liquor. The residue collected was filtered (125 Ø cellulose filter, Whatmann, UK) and left to dry overnight. The leach liquor was analyzed for metal analysis and characterization.

Leaching trials involving a heterotrophic fungal strain (*Aspergillus niger*) were carried out with PCBs and tantalum capacitor scrap at pulp density of 2% (*w*/*v*), particle size of 0.75 mm and varying inoculum conditions (*A. niger* spore and *A. niger* filtrate). Experiments were carried out in 250 mL Erlenmeyer flasks, using a rotary shaker rotating at 150 rpm at 30 °C (Gallenkamp Orbital Shaker—(incubator). Comparative control experiment was also carried out, to maintain similar initial pH of medium, the pH of control experiments was adjusted to 5.6 ± 0.1 using HCl. From previous experiments, high-intensity sampling can contaminate the cultures so to minimize this risk, pH was measured every 5th day and the experiments were performed over 20 days. After 20 days the flasks were harvested to obtain the leached residues and the final leached liquor. The residue collected was filtered (125 Ø cellulose filter, Whatmann, UK) and left to dry overnight. The leach liquor was analyzed for metal analysis and characterization.

## 3. Results

### 3.1. Characterisation of Samples

The chemical analysis of shredded PCBs from spent mobile phones used in this research work showed Cu, Fe, Zn and Ni as major metals, with detectable levels of precious metals such as Au, Ag and Pd, confirming that waste is a rich source of many critical elements and great recycling potential. Around 29% tantalum and high values of Mn and Ag were detected in the tantalum capacitor scrap sample. Table 1 summarizes these concentrations. 

In Table 2 we compare our results with those from previous studies, showing the order of magnitude agreement, despite considerable intra-study variability. 

### 3.2. Leaching Experiments

#### 3.2.1. Leaching with Organic Acids

**Figure 1:**
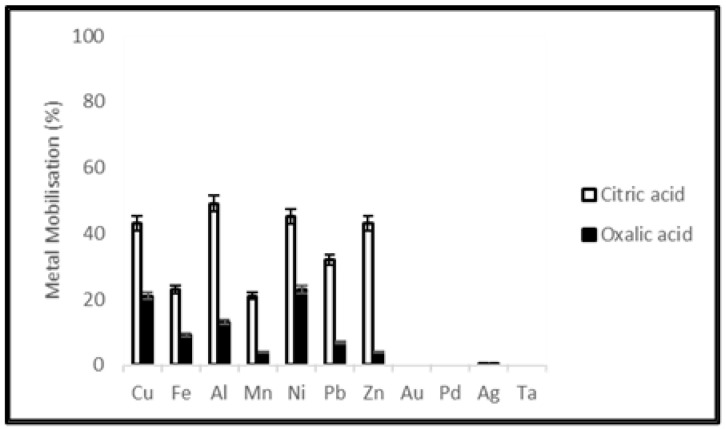
Metal mobilization from PCB scrap after 20 days leaching using citric acid and oxalic acid.

**Figure 2:**
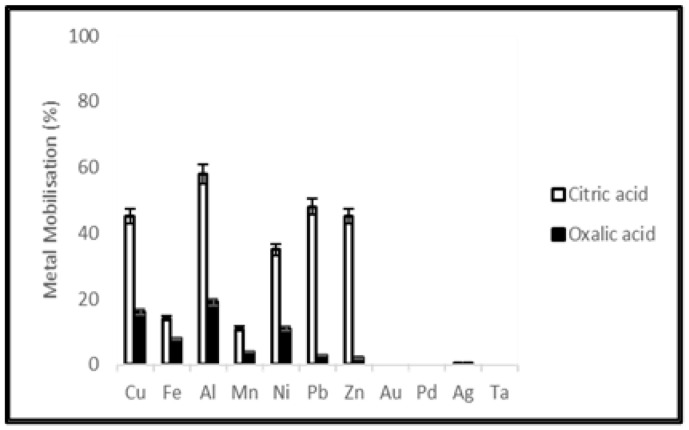
Metal mobilization from tantalum capacitor scrap after 20 days leaching using citric acid and oxalic acid.

Solubility of metals from waste PCBs and tantalum capacitors were studied using citric acid (0.05 M) and oxalic acid (0.05 M). The leaching efficiencies of different metals from PCBs and tantalum capacitor samples after a leaching period of 20 days are shown in Figure 1 and Figure 2. The figures show citric acid as providing better leaching efficiency in mobilizing Cu, Fe, Al, Mn, Ni, Pb and Zn at a level of 45, 14, 58, 11, 35, 48, 45% from PCB samples and 43, 23, 49, 21, 45, 32, 43% from tantalum capacitor samples, respectively.

#### 3.2.2. Leaching with Inorganic Acids

**Figure 3:**
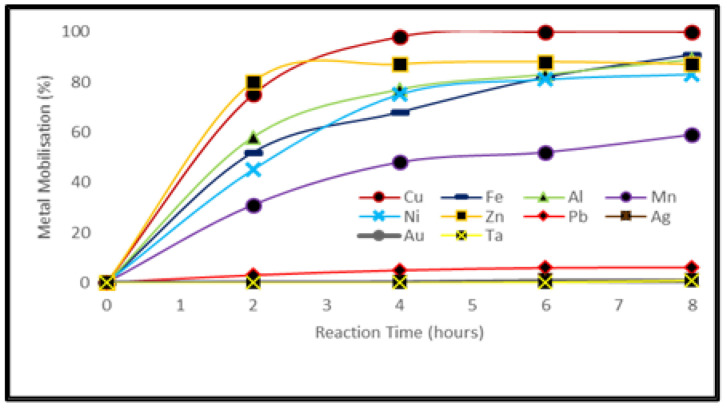
Metal mobilization after treating PCB samples with H_2_SO_4_ and H_2_O_2_ (sample: 10 g, solution: 100 mL, H_2_SO_4_: 2 M, H_2_O_2_: 5 vol %, temp.: 85 °C).

Experiments using H_2_SO_4_ provided optimized conditions and are summarized in Supporting Document Table S2. The optimal conditions were found for a solution with 2 mol/L sulfuric acid and 1.5 mol/L of H_2_O_2_ and a reaction temperature of 85 °C for 4 h. Under these conditions, 100% extraction efficiency of Cu was achieved, whereas Zn, Fe, Al and Ni were leached at a rate of ≈85%. Figure 3 shows the optimization of conditions for metal leaching from PCBs with H_2_SO_4_.

**Figure 4:**
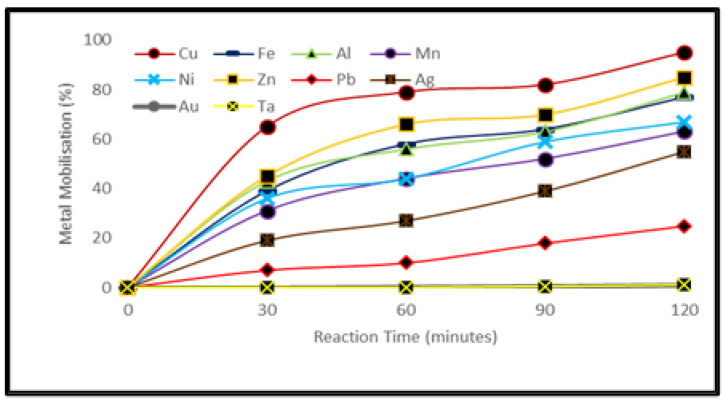
Metal mobilization after treating PCB samples with HNO_3_ (sample: 10 g, solution: 100 mL, HNO_3_: 4 M, temp.: 80 °C).

Due to its strong oxidizing power, HNO_3_ was selected to investigate the dissolution efficiency of metals in PCB waste [34]. The optimal leaching conditions for HNO_3_ were found to be a solution of 4 mol/L HNO_3_ and a reaction temperature of 80 °C for 2 h. Under these conditions, the recovery of Cu was 95%, whereas Zn, Fe, pb and Ni were recovered at ≈80%. It was observed that the extraction efficiency of metals increased considerably when the HNO_3_ concentration was increased from 1–4 M, however, further increase in acid concentration had very little effect to increase metal dissolution. Figure 4 represents the optimal conditions for metal leaching from PCBs with HNO_3_. For ICP-OES analysis see Supporting Documents Table S3.

#### 3.2.3. Microbial Leaching

**Figure 5:**
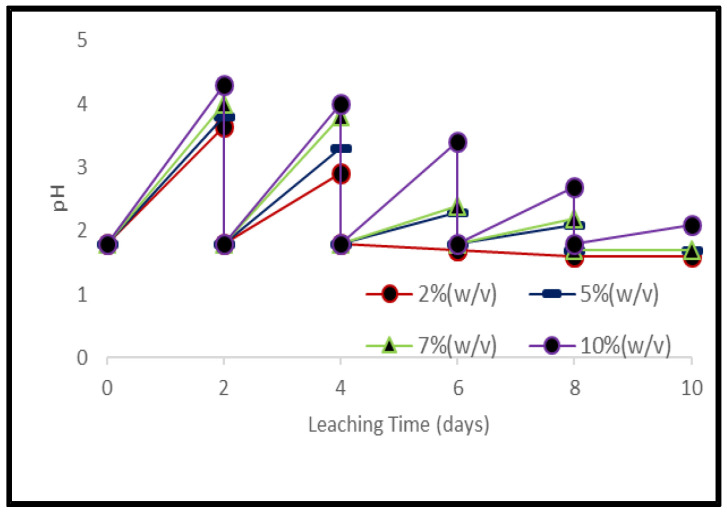
pH variance during bacterial leaching of PCB samples at different pulp densities.

**Figure 6:**
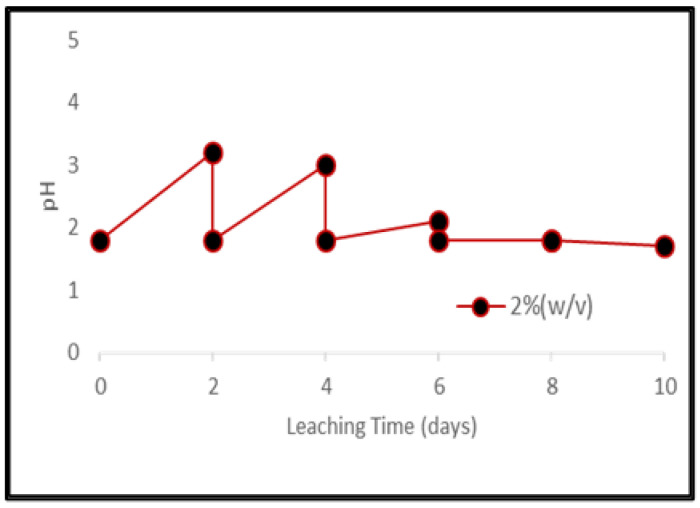
pH variance during bacterial leaching of Ta capacitor samples at pulp density 2% (*w*/*v*).

To avoid the inhibitory effect on bacterial growth due to the toxic nature of electronic waste, the bacterial culture was activated before being used in the microbial studies [35,36]. A rapid increase in pH was observed immediately after adding the grounded samples of PCBs and tantalum capacitor scrap. Higher material load showed higher pH value and lower leaching efficiency. After 4 days, pH for the lower material load (2%) maintained itself, however, for higher material loads, it had to be maintained till the end of the experiment. Figure 5 represents pH variance during microbial leaching at different pulp densities of PCB samples whereas Figure 6 represents pH variation for 2% tantalum capacitor samples during the experiment (Supporting Document Table S4).

**Figure 7:**
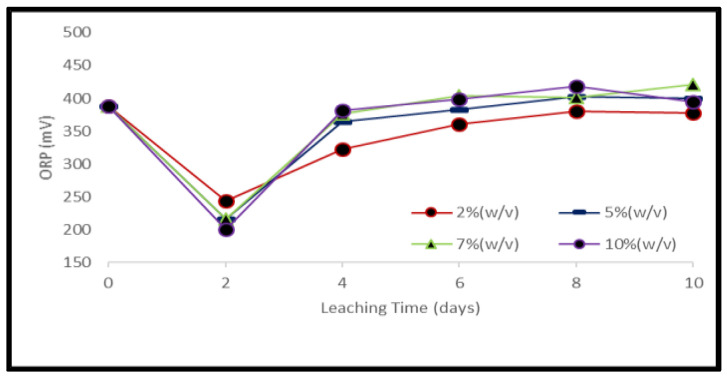
Evolution of ORP during bacterial leaching of PCB samples at different pulp densities.

**Figure 8:**
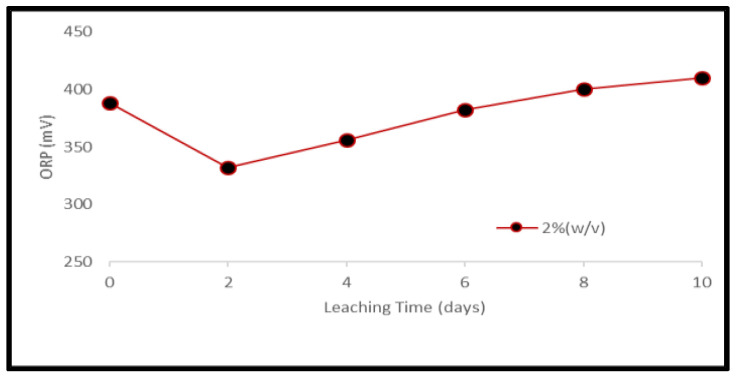
Evolution of ORP during bacterial leaching of Ta capacitor samples at pulp density 2% (*w*/*v*).

**Figure 9:**
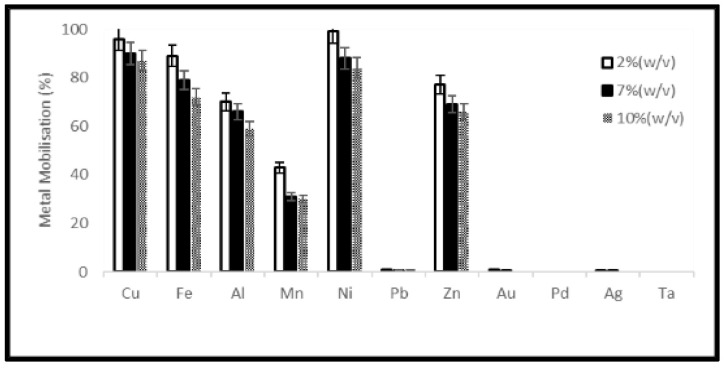
Metal mobilization from PCB scrap after 10 days bacterial leaching varying pulp densities.

**Figure 10:**
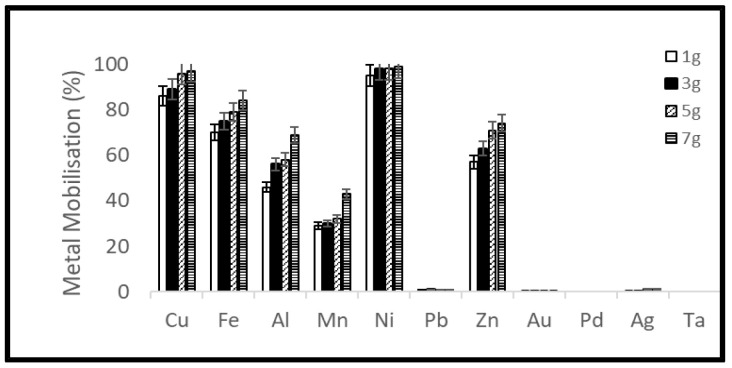
Metal mobilization from PCB scrap after 10 days bacterial leaching varying ferrous iron concentration.

**Figure 11:**
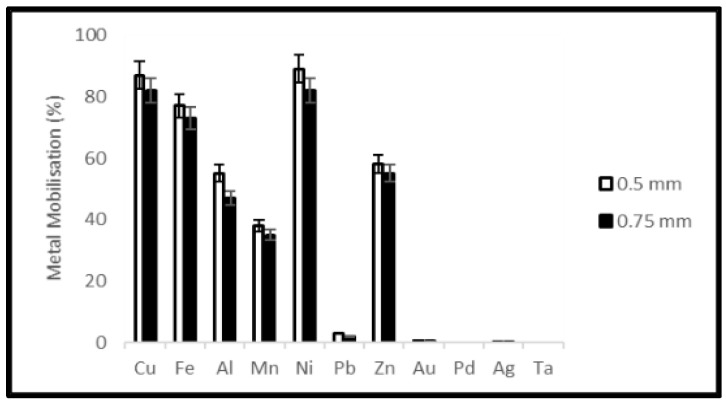
Metal mobilization from PCB scrap after 10 days bacterial leaching varying particle size.

**Figure 12:**
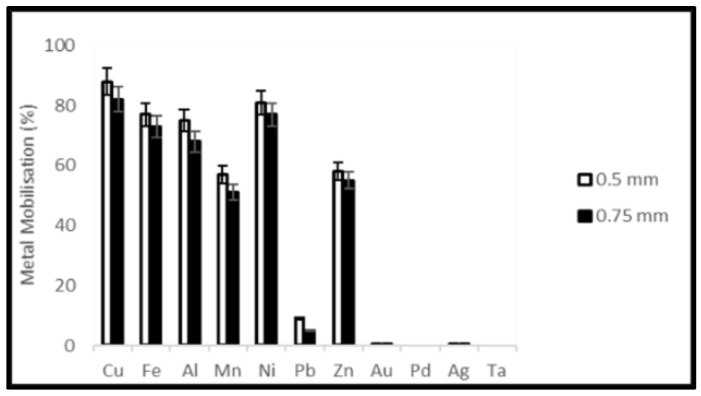
Metal mobilization from Ta capacitor scrap after 10 days bacterial leaching varying particle size.

A reduction of the ORP of the solution was observed soon after the addition of samples which was stronger for the higher material load. On the third day of the experiments, the ORP of the solution started to increase again (Figure 7 and Figure 8). Metal mobilization from PCBs and tantalum capacitor scrap by acidophiles after a 10-day leaching period under different experimental conditions is shown in Figure 9, Figure 10, Figure 11 and Figure 12. A mixed consortium of acidophiles leached almost all base metals at higher rates from both PCB and tantalum capacitor samples while precious and rare metals were leached at negligible amounts.

The microbial leaching profile of metals at different pulp densities is given in Figure 9. Among the base metals, Ni and Cu exhibited maximum leaching efficiency (99 and 96%, respectively) while Fe, Zn, Al and Mn were leached at an efficiency of 89%, 77%, 70%, and 43%, respectively, from PCB samples. The same leaching pattern was observed in the case of tantalum capacitor samples, acidophiles mobilized 92% Cu, 88% Ni, 78% Fe, 77% Al, 70% Zn and 57% Mn. For a detailed analysis see Supporting Documents Tables S5 and S6. 

**Figure 13:**
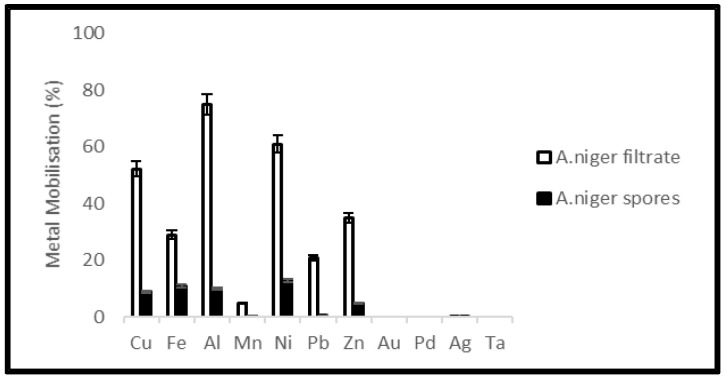
Metal mobilization from PCB scrap after 20 days fungal leaching by *A. niger* spores and filtrate.

**Figure 14:**
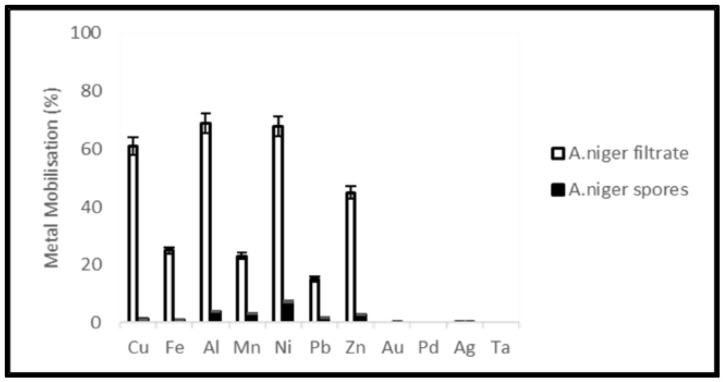
Metal mobilization from Ta capacitor scrap after 20 days fungal leaching by *A. niger* spores and filtrate.

**Figure 15:**
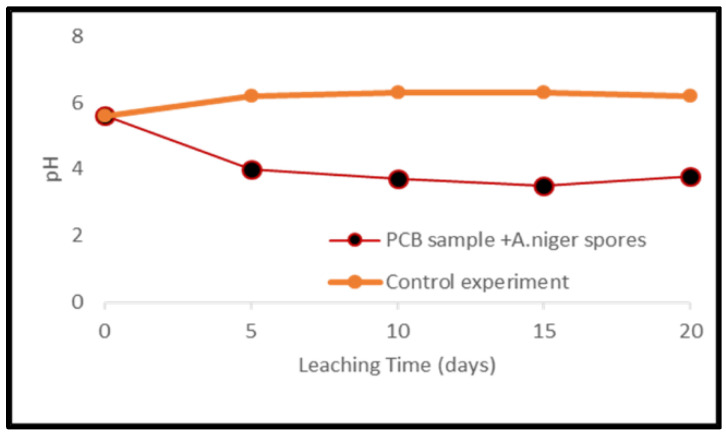
pH variance during fungal leaching of PCB samples.

**Figure 16:**
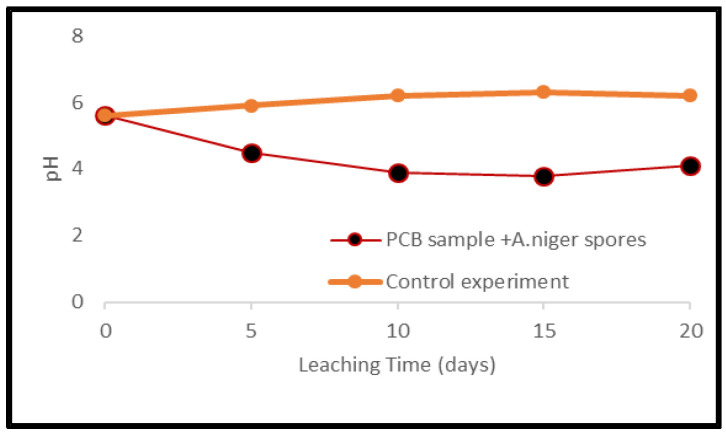
pH variance during fungal leaching of Ta capacitor samples.

Metal mobilization from PCBs and tantalum capacitor scrap after a 20-day leaching period by *A. niger* spores and filtrate is shown in Figure 13 and Figure 14. The pH of the medium over the experimental time is given in Figure 15 and Figure 16. 

Leaching experiments resulted in efficient solubilization of base metals by *A. niger* filtrate. Cu, Fe, Al, Mn, Ni, Pb and Zn were mobilized at an efficiency of 52, 29, 75, 5, 61, 21 and 35% from PCB samples and 61, 25, 69, 23, 68, 15 and 45% from tantalum capacitor samples, respectively. However, *A. niger* spores failed to mobilize metal from samples at any appreciable amount. Precious metals and rare earth metals were also not detected in the leachate. Metal solubility in the control experiments was negligible. The pH profile and acid production during microbial leaching dropped steadily from 5.6 to 3.5 (PCB samples) and 3.8 (tantalum capacitor samples), respectively, after which a gradual increase in the pH profile was observed until the end of the experiments. See Supporting Document Table S7.

From the experimental results, it was found that pulp density, pH, acid concentration, particle size, temperature and reaction time are important operating parameters that control the leaching processes and therefore must be maintained in a certain range for optimum metal leaching from PCBs. Under optimum working conditions, the leaching efficiency of selected metals is shown in Table 3.

## 4. Discussion

### 4.1. Metal Extraction by Organic Acid Leaching

Synthetic organic acids namely citric acid and oxalic acid were selected to conduct a comparative study between chemical and microbial leaching mechanisms. Leaching was terminated after 20 days when the metal concentration in the leachate was relatively constant. Dissolution of the metallic fraction from electronic waste by organic acids involves various mechanisms including acidification and complexation. To compare chemical leaching using commercial citric and oxalic acids with microbial production, the molarity of acid solutions used was decided on from previous studies of their production by the filamentous fungus *A. niger* [36,37,38]. Citric acid gave better leaching efficiency than oxalic acid, however, commercial citric acid showed a lower extraction yield than the equivalent molarity during microbial leaching using *A. niger* filtrate. This study showed that purified and preconcentrated microbial extract could be a viable alternative for metal leaching. 

### 4.2. Metal Extraction by Inorganic Acid Leaching

Sulfuric acid in the presence of H_2_O_2_ forms peroxysulfuric acid (H_2_SO_5_), which acts as a strong oxidizing agent and increases metal dissolution, however, the decomposition of H_2_O_2_ starts at agitation speeds higher than 500 rpm, thus decreasing the dissolution of metals [39]. The following equations describe the chemical dissolution process.

(5) $$2H_{2}O_{2\;}\to 2H_{2}O+O_{2}$$

(6) $$H_{2}SO_{4}+H_{2}O_{2}\to \;H_{2}SO_{5}+H_{2}O$$

(7) $$Cu+H_{2}SO_{5}\;\to Cu^{2+}+SO_{4}^{2-}+H_{2}O$$

Under optimum conditions, H_2_SO_4_ leached Cu from waste PCBs with 100% extraction efficiency whereas Zn, Fe, Al and Ni were leached at a rate of ≈85%. The high corrosion potential of H_2_SO_4_ (−9.8 mV.SCE) is responsible for this behavior [40]. It was observed that an increase in sulfuric acid concentration had little effect on metal dissolution rate and is not suitable from an economic point of view but an increase in temperature from 70–80 °C significantly increased the metal leaching efficiency. Further increase in temperature, however, shows only a slight effect on metal dissolution, the reason being the accelerated rate of H_2_O_2_ decomposition at these higher temperatures.

The application of nitric acid under these conditions gave a recovery of Cu at 95%, whereas Zn, Fe, Pb and Ni were recovered at ≈80%. It was observed that the extraction efficiency of metals increased considerably when nitric acid concentration was increased from 1–4 M. At concentrations higher than 4 M, the H^+^ was saturated and a further increase in acid concentration had very little effect on metal dissolution efficiency. However, the leaching results demonstrate that inorganic acids due to their fast reaction rates are relatively more efficient compared to the leaching by more eco-friendly organic acids.

### 4.3. Metal Extraction by Acidophilic Bacterial Strains

Due to the non-sulfidic nature of electronic waste, it was speculated that metal solubilization from samples followed both direct and indirect leaching mechanisms involving the biogenic formation of leaching agents [19,41]. Acidophilic autotrophs generally oxidize ferrous (Fe^2+^) to ferric iron (Fe^3+^) and elemental sulfur (S^0^) to sulfuric acid (H_2_SO_4_), and this biogenic production of ferric iron (redoxolysis) and sulfuric acid (acidolysis) results in the dissolution of metals from PCBs and tantalum capacitor scrap. The alkalinity of electronic waste and oxidation of ferrous to ferric iron increased the pH of the medium, consequently decay in ORP was observed. Variation in ORP and Fe^2+^ concentration contributes significantly to bacterial activity and subsequently metal leaching efficiency. A higher value of ORP encourages interaction between bacteria and electronic waste for metal solubilization [42], which can be attained with a steady pH profile. The pH of the growth medium is another important parameter that controls the growth and activity of acidophilic microorganisms. The growth of microorganisms is usually initiated at a very low pH (not higher than 3.0) and as the growth continues, the pH of the medium no longer affects the bacterial activity [43]. Metal dissolution through redoxolysis and acidolysis mechanisms is a cyclic and acid-consuming process. Consequently, the alkalinity of the source material decreased, and a steady pH profile was achieved. For a higher material load, a prolonged time was required to show a steady pH profile. Formation of jarosite was observed during the microbial leaching experiment which dominated in experiments with higher material load generating high pH. Studies show that ferric iron precipitation hinders the metal leaching process [44] by reducing the content of dissolved iron in the culture medium [25]. This suggests that pulp density plays an important role in metal solubilization from electronic waste, high pulp density has a negative effect on the productivity of microorganisms due to the toxic effect of metallic and non-metallic compounds contained in electronic waste [24] or oxygen transfer limitation [19]. A pre-cultivation strategy can resolve the problem. Although the results demonstrate that a lower concentration of samples is more effective in metal dissolution as compared to a high concentration, the adaptability of microbial consortia after succession from low to high pulp density while showing high leaching efficiencies [45] enables the use of high metal concentrations while designing the full-scale process. The microbial leaching results verify that the rate of leaching also depends upon the particle size of the sample. Smaller particle size means greater surface area thus the contact area of microbial cells increases leading to a higher yield of metals without a change in the mass of the sample. In this study, 0.75 mm particle size is considered the optimum particle size for metal leaching, albeit a relative increase in the leaching efficiency of base metals was observed when particle size decreased from 0.75 mm to 0.5 mm, however, over-crushing and excessive sieving results in a loss on comminution (LoC) or accumulation of the material on the sieve [46]. An increase in particle size from 0.5 mm to 0.75 mm shows a decrease in leaching rate by a factor of 1.2. A balance between the crushing process and particle size is maintained, hence microbial leaching experiments with a particle size greater than 0.75 mm were not studied. The significant factors behind this decision were leaching efficiency profile, LoC and manual labor. In the control experiments, very low concentrations of Cu and Fe (3.2 and 1%, respectively) were detected, and it was presumed that the mobilization of metals, in this case, was due to the exposure to H_2_SO_4_ for pH adjustment.

Metal solubilization through acidophiles is an electrochemical process based on oxidation-reduction reactions. Metals with lower standard electrode potential oxidize first [40] as compared to metals with higher standard electrode potential. Moreover, metal mobilization is highly influenced by the solubility of metals as in the case of Pb. Pb was not detected in the leachate and it was speculated that Pb was precipitated as PbSO_4_ in the form of white precipitates observed in the experimental flasks [23]. Similarly, the precious metals and tantalum were also not detected in the leachate. 

### 4.4. Metal Extraction by Fungal Leaching

Due to the ability to remove toxic organic compounds [39] in samples and diversity in metabolic production, *A. niger* was tested for leaching of metals from PCB and tantalum capacitor scrap. Citric, oxalic, malic and gluconic acids are the most abundant acids produced by *A. niger* for carrying out the leaching process [47,48]. Metal mobilization by *A. niger* spores as well as filtrate was studied. *A. niger* filtrate showed better results and mobilized base metals Cu, Fe, Al, Ni and Zn at a rate of 52, 29, 75, 51 and 35% from PCBs and 65, 25, 69, 68 and 45%, respectively, from tantalum capacitor scrap.

From the results, it is obvious that direct growth in the presence of electronic waste is not recommended. Therefore, microorganisms should be grown in the absence of electronic waste and the metabolites formed should be used for metal leaching. The use of metabolites for microbial leaching has multiple advantages, biomass as well as waste material is not contaminated and can be recycled and used again, the acid formation can be optimized, and a higher material load can be applied. No doubt the use of live culture is more interesting in metal recovery since microorganisms are functional and continuous production of metabolites is available but factors that hinder the microorganism’s growth should be addressed. Although the metal toxicity in the case of tantalum capacitor samples is lower compared to PCB samples, the presence of very high levels of manganese in tantalum capacitor scrap reduces the accumulation of citric acid significantly. In a manganese-deficient medium, the enzyme isocitrate dehydrogenase is unable to catalyze the oxidative decarboxylation of isocitrate to alpha-ketoglutarate (in the Krebs cycle) and citric acid is accumulated in the medium, however, the presence of manganese releases isocitrate dehydrogenase into the medium, and citrate is converted to organic acids such as succinate, fumarate, malate, etc. and reduces the accumulation of citric acid [41].

Another factor that significantly affects the leaching efficiency of metal-using fungal strains is the cultivation period, with some authors incubating fungi for a prolonged period [38] resulting in high leaching efficiencies. The pH profile during leaching experiments is directly related to acid production and in experiments with *A. niger* spores, a steady decrease in profile was observed up to day 15, after which increases were observed. The pH drop during fungal growth was due to the production of organic acids [34] with a maximum at day 15. This trend shows a decrease in acid production which could be due to the presence of metal ions and the formation of insoluble metal oxalates through an intermediate solubilization process [27].

### 4.5. Comparative Evaluation of Chemical and Microbial Leaching of Metals 

Experimental results demonstrated that pH, pulp density, reaction temperature and time are all important parameters in three types of leaching. According to Pant et al. [46], chemical leaching leads to higher resource leaching as compared to microbial leaching. However, the leaching trials in this study prove that microbial leaching has almost the same metal leaching potential as chemical leaching. Precious and rare metals were leached at negligible amounts in both chemical and microbial leaching experiments apart for NHO_3_, which shows a great ability to solubilize silver. The reaction time for chemical leaching is quite low as compared to microbial leaching but requires high temperature and energy. Even though chemical leaching is always allied with the ecological issues resulting from the corrosive nature and chemical toxicity of the leaching solution, the processes are based on established stoichiometry, therefore, the degree of ambiguity is low as compared to biological methods [47,49]. The main constraint in microbial leaching is the optimization of the process which needs a deeper understanding of the mechanism and kinetics to define optimal operating parameters. On the other hand, it is potentially a more economic and environmentally sustainable approach [48], particularly when considering multiple step processing [50,51].

## 5. Conclusions

The result of the study reveals that microbial leaching and chemical leaching (inorganic acids) in terms of metal extraction from electronic waste are equally efficient techniques. However, leaching with organic acids was less efficient compared to microbial leaching thereby proving that secondary metabolites produced by the fungal strains also add to the leaching process. Metal mobilization by *A. niger* spores and filtrate was also studied; microbial extract being more effective can be a feasible and sustainable alternate for metal leaching. Although microbial leaching requires a longer operational period compared to chemical leaching, its ecological advantages cannot be underestimated. Both approaches have their limitations and a “one-fits all” technique to recover valuable metals from e-waste is still elusive. The microbial leaching techniques studied did prove potent in extracting base metals but were not capable of targetting the rare metals identified. This may be the heterogeneity of waste and low absolute concentrations and multiple processes are recommended for rare and critical metal recovery. Development in bio genomics may prove useful in overcoming current microbial limitations.

The experimental results of this study provide promising indicators for upscaling and show the practicability of microbial leaching of electronic scrap. For the selected acidophilic bacterial strains, the possibility of contamination is minimal, due to the acidic environment and absence of a carbon source, meaning aseptic conditions were not required and upscaling is therefore easier. Pre-growth strategy can solve issues of metal toxicity in electronic waste and acidophiles are known for their adaptability and versatility to inhabit environments with high metal concentrations. The microorganisms studied grow well between 25–30 °C, allowing development for locations with climatic conditions in this range of ambient air instead of a fixed temperature leaching process, thereby significantly reducing the electricity cost of a scaled-up process. 

## Figures and Tables

**Table 1:** Metal content analysis of waste PCBs and Ta capacitors.

	PCBs (Mobile) Microwave Digestion without HF				Ta. Capacitors Microwave Digestion with HF			
	Particle Size				Particle Size			
	0.75		0.5		0.75		0.5	
Base Metals (g/Kg)	Average	SD	Average	SD	Average	SD	Average	SD
Cu	311.6	5.32	267.6	4.87	53.5	1.51	66.3	1.55
Fe	50.6	0.87	17.9	0.41	4.7	0.02	12.2	0.04
Al	8.7	0.34	10.6	0.39	3.9	0.01	4.6	0.01
Mn	13.5	0.51	8.7	0.33	82.3	1.91	53.9	1.72
Ni	22.3	1.23	30.17	1.51	14.3	0.43	24.3	0.54
Pb	10.5	0.76	8.9	0.41	14	0.81	14.7	0.77
Zn	35.1	1.59	25.4	1.38	6.2	0.24	8.8	0.28
**Precious Metals (g/Kg)**								
Au	0.4	0	0.9	0	0.05	0	0.06	0
Pd	0.1	0	0.1	0	0.2	0	0.1	0
Ag	2.2	0.02	1.4	0.01	14.9	0.21	19.9	0.27
**Critical Metal (g/Kg)**								
Ta	0.03	0	0.03	0	292.5	2.32	292.5	2.32

**Table 2:** Comparison of metal content analysis of waste PCBs and Ta capacitors (VICs) reported in different studies with the present study.

Sample	Base Metals (g/Kg)							Precious Metals (g/Kg)			Critical Metal (g/Kg)	References
	Cu	Fe	Al	Mn	Ni	Pb	Zn	Au	Pd	Ag	Ta	
PCBs from spent mobile phones	311.6	50.6	8.7	13.5	22.3	10.5	35.1	0.4	0.1	2.2	0.03	Present Study
	378.1	48.5	6.1	NR	25.4	12.3	18.2	0.9	NR	0.5	NR	[29]
	344.9	105.7	2.6	NR	26.3	18.7	59.2	NR	NR	2.1	NR	[30]
	566.8	2.4	14.2	NR	NR	NR	2.2	0.2	0.1	1.0	NR	[31]
	360.0	10.5	6.7	NR	8.6	12.1	8.0	0.1	0.6	0.3	NR	[32]
Ta Capacitor (Old and new)	53.5	28.7	3.9	82.3	14.3	14.0	6.2	0.05	0.2	14.9	292.5	Present Study
	38	38	21.1	31.8	21.7	1.3	2.8	0.01	ND	32	484	[33]
	NR	NR	NR	175	NR	NR	NR	NR	NR	33	442	[25]

**Table 3:** Optimum metal leaching conditions from PCBs from this study.

	Leaching Agent	Acid Conc. (M)	pH	Particle Size (mm)	Pulp Density (%)	Temp (°C)	Time	Leaching Efficiency (%)										
								Cu	Fe	Al	Mn	Ni	Pb	Zn	Au	Pd	Ag	Ta
Organic Acids	Citric Acid	0.05	-	0.75	10	30	20 days	45	14	58	11	35	48	45	0	0	0.2	0
	Oxalic Acid	0.05	-	0.75	10	30	20 days	16	8	19	4	11	3	2	0	0	0	0
Inorganic Acids	Sulfuric Acid	2	-	0.75	10	85	4 h	98	68	77	48	75	5	87	0.1	0	0.4	0
	Nitric Acid	4	-	0.75	10	80	2 h	95	77	79	63	67	25	85	1.1	0	55	1.3
Microorganisms	Mixed Consortium (*A. ferrooxidans*, *L. ferrooxidans* and *A. thiooxidan*)	-	1.8	0.75	2	30	10 days	96	89	70	43	99	0.8	77	0.8	0	0	0
	*A. niger* (spores)	-	5.6	0.75	2	30	10 days	9	11	10	0.4	13	0.6	5	0	0	0	0
	*A. niger* (filtrate)	-	5.6	0.75	2	30	10 days	52	29	75	5	61	21	35	0	0	0.2	0

## Data Availability

Data can be made available upon reasonable request.

## Supplementary Materials

The following supporting information can be downloaded at: https://www.mdpi.com/article/10.3390/ijerph191610006/s1, Table S1: Chemical composition of waste PCBs and tantalum capacitors (VICs) without HF precious metals; Table S2: Leaching with organic acids; Table S3: Leaching with inorganic acids; Table S4: Bacterial leaching of PCBS varying pulp density; Table S5: Bacterial leaching of PCBS varying ferrous iron concentration; Table S6: Bacterial leaching of PCBS and tantalum capacitor scrap varying particle size; Table S7: Fungal leaching of metals by *A*. *niger* spores and filtrate.
