# Supplementary material for: Chemical and Microbial Leaching of Valuable Metals from PCBs and Tantalum Capacitors of Spent Mobile Phones
Source: Int J Environ Res Public Health. 2022 Aug 13;19(16):10006. doi: 10.3390/ijerph191610006 (PMC9408593; doi:10.3390/ijerph191610006)
Supplement: Supplementary file 1 [file ijerph-19-10006-s001.zip › ijerph-1818908-supplementary.pdf]

Supporting Documents:

Chemical and Microbial Leaching of Valuable Metals from PCBs and Tantalum Capacitors of Spent Mobile Phones

Asma Sikander [1,2], Steven Kelly [3], Kerstin Kuchta [4], Anika Sievers [1], Thomas Willner [1] and Andrew S. Hursthouse [2,*]

[1] Department of Process Engineering, Hamburg University of Applied Sciences, Ulmenliet 20, 21033 Hamburg, Germany

[2] School of Computing, Engineering & Physical Sciences, University of the West of the Scotland, Scotland PA1 2BE, UK

[3] School of Health Life Sciences, University of the West of Scotland, Scotland G72 0LH, UK

[4] Institute for Environmental Engineering and Energy Economics, TUHH—Hamburg University of Technology, D-21079 Hamburg, Germany

* Correspondence: andrew.hursthouse@uws.ac.uk; Tel.: +44-1418483213

S-1. Chemical composition of waste PCBs and tantalum capacitors (VICs) without HF

Precious Metals

Sample	Au (208.207 nm)			Pd (265.945 nm)			Ag (328.068 nm)			Ta (263.558 nm)		
	mg/L	g/kg	Avg. (g/kg)	mg/L	g/kg	Avg. (g/kg)	mg/L	g/kg	Avg. (g/kg)	mg/L	g/kg	Avg. (g/kg)
PCB-0.75a	1.61	0.40	0.4	0.08	0.02	0.00	9.4	2.35	2.2	0.1	0.03	0.02
PCB-0.75b	1.47	0.37		0.06	0.02		8.4	2.10		0.09	0.02	
PCB-0.50a	4.13	1.03	1.0	0.04	0.01	0.00	7.51	1.88	1.4	0.07	0.02	0.02
PCB-0.5b	3.59	0.90		0.05	0.01		3.38	0.85		0.1	0.03	
PCB-0.25a	4.56	1.14	1.2	0.13	0.03	0.00	8.51	2.13	1.8	0.04	0.01	0.01
PCB-0.25b	4.69	1.17		0.1	0.03		5.5	1.38		0.07	0.02	
Cap-0.75	1.01	0.51	-	0.01	0.01	-	29.9	14.95	-	0.32	0.16	-
Cap-0.5	1.21	0.61	-	0.02	0.01	-	39.9	19.95	-	0.22	0.11	-

S-2. Leaching with organic acids

Sample	Cu (327.395 nm)		Fe (261.382 nm)		Al (394.401 nm)		Mn (257.610 nm)		Ni (232.138 nm)		Pb (283.305 nm)		Zn (206.200 nm)		Au (208.207 nm)		Pd (340.458 nm)		Ag (328.068 nm)		Ta (268.517 nm)	
	mg/L	metal leached (%)	mg/L	metal leached (%)	mg/L	metal leached (%)	mg/L	metal leached (%)	mg/L	metal leached (%)	mg/L	metal leached (%)	mg/L	metal leached (%)	mg/L	metal leached (%)	mg/L	metal leached (%)	mg/L	metal leached (%)	mg/L	metal leached (%)
**Citric Acid Leaching**																						
PCBs	14104.6	45.27	710.90	14.05	510.31	58.66	149.67	11.09	788.09	35.34	500.89	47.70	1591.0	45.33	0.01	0	u	0	0.51	0.23	u	0
Ta.Cap	2307.89	43.14	667.13	23.24	189.86	48.68	1743.6	21.19	645.65	45.15	443.45	31.68	267.34	43.12	u	0	u	0	3.1	0.21	u	0
**Oxalic Acid Leaching**																						
PCBs	4869.7	15.63	401.71	7.94	168.52	19.37	59.43	4.40	250.81	11.25	34.87	3.32	69.63	1.98	u	0	u	0	0.1	0.05	u	0
Ta.Cap	1122.45	20.98	272.01	9.48	49.61	12.72	321.54	3.91	329.45	23.04	96.61	6.90	26.01	4.20	u	0	u	0	u	0	u	0

S-3. Leaching with inorganic acids

Sample	Cu (327.395 nm)		Fe (261.382 nm)		Al (394.401 nm)		Mn(257.610 nm)		Ni (232.138 nm)		Pb (283.305 nm)		Zn (206.200 nm)		Au (208.207 nm)		Pd (340.458 nm)		Ag (328.068 nm)		Ta (268.517 nm)	
	mg/L	metal leached (%)	mg/L	metal leached (%)	mg/L	metal leached (%)	mg/L	metal leached (%)	mg/L	metal leached (%)	mg/L	metal leached (%)	mg/L	metal leached (%)	mg/L	metal leached (%)	mg/L	metal leached (%)	mg/L	metal leached (%)	mg/L	metal leached (%)
**Sulfuric Acid Leaching**																						
PCBs-0.75	30399.94	97.56	3464.89	68.48	670.11	77.02	651.85	48.29	1680.32	75.35	49.33	4.70	3071.65	87.51	0.05	0.13	u	0	0.91	0.41	u	0
**Nitric Acid Leaching**																						
PCBs-0.75	29709.62	95.35	3909.76	77.27	685.11	78.75	856.34	63.43	1498.45	67.20	267.21	25.45	2991.65	85.23	0.45	1.13	u	0	121.9	55.41	0.04	1.30

S-4. Bacterial leaching of PCBs varying pulp density

Sample	Cu (224.700 nm)		Fe (259.940 nm)		Al (309.271 nm)		Mn (260.568 nm)		Ni (230.078 nm)		Pb (283.305 nm)		Zn (210.442 nm)		Au (208.207 nm)		Pd (340.458 nm)		Ag (338.068 nm)		Ta (263.558 nm)	
	mg/L	metal leached (%)	mg/L	metal leached (%)	mg/L	metal leached (%)	mg/L	metal leached (%)	mg/L	metal leached (%)	mg/L	metal leached (%)	mg/L	metal leached (%)	mg/L	metal leached (%)	mg/L	metal leached (%)	mg/L	metal leached (%)	mg/L	metal leached (%)
**Pulp Density 2%**																						
PCBs	6025.36	96.68	899.1	88.84	121.9	70.06	120.9	43	440.66	98.8	1.69	0.80	541.22	77.10	0.07	0.88	u	0	0.05	0.1	u	0
**Pulp Density 7%**																						
PCBs	19700.12	90.32	2788.67	78.73	399.99	65.68	289.78	30.66	1372.88	87.95	4.59	0.62	1703.64	69.34	0.05	0.18	u	0	0.1	0.06	u	0
**Pulp Density 10%**																						
PCBs	26990.65	86.62	3659.34	72.32	509.88	58.61	399.69	29.61	1870.77	83.89	8.7	0.83	2304.9	65.67	u	0	u	0	0.14	0.06	u	0

S-5. Bacterial leaching of PCBs varying ferrous iron concentration

Sample	Cu (224.700 nm)		Fe (259.940 nm)		Al (309.271 nm)		Mn(260.568 nm)		Ni (230.078 nm)		Pb (283.305 nm)		Zn (210.442 nm)		Au (208.207 nm)		Pd (340.458 nm)		Ag (338.068 nm)		Ta (263.558 nm)	
	mg/L	metal leached (%)	mg/L	metal leached (%)	mg/L	metal leached (%)	mg/L	metal leached (%)	mg/L	metal leached (%)	mg/L	metal leached (%)	mg/L	metal leached (%)	mg/L	metal leached (%)	mg/L	metal leached (%)	mg/L	metal leached (%)	mg/L	metal leached (%)
**Ferrous Iron Concentration 1g**																						
PCBs	5190.09	83.28	658.66	65.08	78.65	45.20	81.44	30.16	431.65	96.78	1.75	0.83	382.09	54.43	0.04	0.5	u	0	0.15	0.34	u	0
**Ferrous Iron Concentration 3g**																						
PCBs	5379.09	86.31	779.09	76.99	101.01	58.05	82.01	30.37	409.76	91.87	1.81	0.86	399.01	56.84	0.03	0.38	u	0	0.21	0.48	u	0
**Ferrous Iron Concentration 5g**																						
PCBs	5781.99	92.78	802.98	79.35	103.45	59.45	88.99	32.96	425.77	95.46	1.79	0.85	419.65	59.78	0.03	0.40	u	0	0.28	0.64	u	0
**Ferrous Iron Concentration 7g**																						
PCBs	5899.44	94.66	831.45	82.16	109.99	63.21	113.09	41.89	430.21	96.46	1.77	0.84	439.68	62.63	0.04	0.50	u	0	0.31	0.70	u	0

S-6. Bacterial leaching of PCBs and tantalum capacitor scrap varying particle size

Sample	Cu (327.395 nm)		Fe (261.382 nm)		Al (394.401 nm)		Mn (257.610 nm)		Ni (232.138 nm)		Pb (283.305 nm)		Zn (206.200 nm)		Au (208.207 nm)		Pd (340.458 nm)		Ag (328.068 nm)		Ta (268.517 nm)	
	mg/L	metal leached (%)	mg/L	metal leached (%)	mg/L	metal leached (%)	mg/L	metal leached (%)	mg/L	metal leached (%)	mg/L	metal leached (%)	mg/L	metal leached (%)	mg/L	metal leached (%)	mg/L	metal leached (%)	mg/L	metal leached (%)	mg/L	metal leached (%)
**Particle Size 0.5mm**																						
PCBs	5409.54	86.80	782.19	77.29	96.76	55.61	102.67	38.03	397.09	89.03	6.00	2.86	411.09	58.56	0.05	0.60	u	0	0.09	0.20	u	0
Ta.Cap	941.99	88.04	446.98	77.87	58.98	75.62	945.99	57.47	235.77	82.44	26.79	9.57	71.65	57.78	u	0	u	0	0.66	0.23	u	0
**Particle Size 0.75mm**																						
PCBs	5098.45	81.81	739.02	73.03	81.92	47.08	96.03	35.57	369.67	82.89	5.03	2.40	390.08	55.57	0.03	0.38	u	0	0.02	0.05	u	0
Ta.Cap	891.65	83.33	423.08	73.71	53.02	67.97	837.6	50.89	220.04	76.94	16.06	5.74	68.67	55.38	u	0	u	0	0.16	0.05	u	0

S-7. Fungal leaching of metals by *A.*Niger spores and filtrate

Sample	Cu (327.395 nm)		Fe (261.382 nm)		Al (394.401 nm)		Mn(257.610 nm)		Ni (232.138 nm)		Pb (283.305 nm)		Zn (206.200 nm)		Au (208.207 nm)		Pd (340.458 nm)		Ag (328.068 nm)		Ta (268.517 nm)	
	mg/L	metal leached (%)	mg/L	metal leached (%)	mg/L	metal leached (%)	mg/L	metal leached (%)	mg/L	metal leached (%)	mg/L	metal leached (%)	mg/L	metal leached (%)	mg/L	metal leached (%)	mg/L	metal leached (%)	mg/L	metal leached (%)	mg/L	metal leached (%)
**Fungal leaching by *A.Niger* spores**																						
PCBs	579.40	9.30	114.34	11.30	33.09	19.02	1.2	0.44	57.87	12.98	1.4	0.67	34.14	4.86	u	0	u	0	0.02	0.05	u	0
Ta.Cap	14.01	1.31	5.99	1.04	2.95	3.78	51.09	3.10	21.76	7.61	4.99	1.78	3.46	2.79	u	0	u	0	0.19	0.06	u	0
**Fungal leaching by *A.Niger* filtrate**																						
PCBs	3219.65	51.66	300.66	29.71	129.91	74.66	12.9	4.78	276.1	61.91	44.65	21.26	243.40	34.67	u	0	u	0	0.12	0.27	u	0
Ta.Cap	651.77	60.91	145.99	25.43	54.54	69.92	376.09	22.85	200.06	69.95	42.55	15.20	55.99	45.15	u	0	u	0	0.5	0.17	u	0
